# Identification of Psychological Dysfunctions and Eating Disorders in Obese Women Seeking Weight Loss: Cross-Sectional Study

**DOI:** 10.1155/2014/356289

**Published:** 2014-03-11

**Authors:** Maude Panchaud Cornut, Jennifer Szymanski, Pedro Marques-Vidal, Vittorio Giusti

**Affiliations:** ^1^Service of Endocrinology, Diabetes and Metabolism, University Hospital CHUV, rue du Bugnon, 1011 Lausanne, Switzerland; ^2^Institute of Social and Preventive Medicine, University Hospital CHUV, rue du Bugnon, 1011 Lausanne, Switzerland; ^3^Département de Médecine, Hôpital Intercantonal de la Broye, 1470 Estavayer-le-Lac, Switzerland

## Abstract

*Objective*. The aim of this study is to analyse associations between eating behaviour and psychological dysfunctions in treatment-seeking obese patients and identify parameters for the development of diagnostic tools with regard to eating and psychological disorders. *Design and Methods*. Cross-sectional data were analysed from 138 obese women. Bulimic Investigatory Test of Edinburgh and Eating Disorder Inventory-2 assessed eating behaviours. Beck Depression Inventory II, Spielberger State-Trait Anxiety Inventory, form Y, Rathus Assertiveness Schedule, and Marks and Mathews Fear Questionnaire assessed psychological profile. *Results*. 61% of patients showed moderate or major depressive symptoms and 77% showed symptoms of anxiety. Half of the participants presented with a low degree of assertiveness. No correlation was found between psychological profile and age or anthropometric measurements. The prevalence and severity of depression, anxiety, and assertiveness increased with the degree of eating disorders. The feeling of ineffectiveness explained a large degree of score variance. It explained 30 to 50% of the variability of assertiveness, phobias, anxiety, and depression. *Conclusion*. Psychological dysfunctions had a high prevalence and their severity is correlated with degree of eating disorders. The feeling of ineffectiveness constitutes the major predictor of the psychological profile and could open new ways to develop screening tools.

## 1. Introduction

The prevalence of obesity has increased markedly worldwide during the past 20 years [1]. Among US adults, approximately 127 million are overweight, 60 million are obese, and 9 million have morbid obesity (body mass index (BMI) > 40 kg/m^2^) [2–4].

This increase has a major impact on public health and on health care costs because of the raise of obesity-related diseases [4]. Further, and despite more than $30 billion spent per year on weight-reduction programs [4–6], their efficacy has not increased accordingly [7]. A recent review suggested that the standard conservative treatments (diet, physical activity, cognitive-behavioural therapy, and drugs) are ineffective in the long term in 95% of the patients [8]. After diet alone, 75% of patients regain most of their weight [9] and the addition of behavioural treatments only modestly improves the results [10]. Bariatric surgery is currently the only treatment achieving a sufficient and durable weight loss [11, 12]; still, follow-up studies show that a number of patients present a weight regain as early as 1 to 2 years after surgery [13, 14].

One of the major reasons for the treatments' ineffectiveness is the large prevalence of eating disorders in obese patients trying to lose weight [15], namely, binge eating disorder (BED) [16]. The impact of weight loss programs on the reduction of BED is low and actually tends to exacerbate the severity of BED and obesity [17, 18]. Specific treatments including psychological support are essential in those patients to improve long term results and to escape from weight cycling [19–22]. Interestingly, most patients with BED start binge eating prior to the onset of dieting. The eating disorder therefore seems to be the* primum movens* leading to weight gain [23, 24]. Other psychological dysfunctions such as depression are also frequent among obese subjects [25]. Moreover, obese patients with BED present a higher prevalence and/or severity of most psychological dysfunctions than obese patients without BED [26–32]. The detection of psychological dysfunctions in obese patients is essential as these are associated with lower weight self-efficacy and limited weight loss [20, 33]. This evidence suggests that the identification of potential psychological dysfunctions is a very important step in the assessment of an obese patient, as are the detecting potential cardiovascular and metabolic comorbidities. Unfortunately, the implementation of psychological assessment is complex and time consuming and requires the use of specific questionnaires by psychologists.

Despite the fact that a number of studies already showed that binge eating in obese women is a marker for greater psychiatric morbidity, no data is available concerning potential predictive factors for the identification of psychological distress in patients who suffer from eating disorders.

Thus, the aim of this study was to analyse the associations between eating behaviour and psychological dysfunctions in obese patients searching weight loss and to identify possible predictive parameters for future development of diagnostic tools in the field of eating and psychological disorders.

## 2. Materials and Methods 

### 2.1. Patient's Sampling

This work was approved by the Ethical Committee of Lausanne University Medicine School and was conducted at the Outpatient Obesity Clinic of the University Hospital of Lausanne, Switzerland. Inclusion criteria were female gender, willingness to lose weight, and agreeing to participate; as men represent less than 5% of our Obesity Clinic, it was decided to exclude them as it would be very difficult to obtain an adequate sample size. The current use of psychotropic medication was an exclusion criterion. Overall, one hundred and fifty women trying to lose weight and to control food compulsion accepted to participate.

### 2.2. Anthropometric Measurements and Weight History

Body weight was measured in kg with a Detecto scale with a precision of 0.2 kg; height was measured in cm with a stadiometer with a precision of 0.5 cm. For the weight the clothes and shoes were left off and for the height the shoes and socks were left off. For each parameter only one measurement was taken. BMI was calculated as weight/height squared (kg/m^2^). Waist circumference was taken at the smallest standing horizontal circumference between the ribs and the iliac crest using a TEC anthropometric tape (Rollfix, Hoechst Mass, Germany). Three measurements were taken with the criterion that difference between the measurements had to be less than 2 cm apart and an average of these three values was calculated. Additional measurements were taken when needed until this criterion was fulfilled.

A specific case history was taken in order to estimate weight history and fluctuation during the patient's life. The participation in organised weight loss programs defined as a diet following a defined program through a nutritionist or an organisation and the number of intentional weight loss attempts were collected. The previous use of weight loss drugs was also registered. The presence of a weight cycling syndrome (WCS), defined as at least 3 weight reductions of ≥5 kg with a subsequent regain of ≥50% of the weight loss, was also assessed [34, 35].

### 2.3. Eating Behaviours and Eating Disorders

The eating behaviours over the last six months before evaluation were assessed by the use of a clinical specific interview and two specific questionnaires: the Bulimic Investigatory Test of Edimburgh (BITE) [36] and the Eating Disorder Inventory-2 (EDI-2) [37, 38].

Regarding the BITE scales the score used was proposed previously by Henderson and Freeman [36]: symptom score was divided in three groups: high (≥20, indicating presence of binge eating), medium (10–19, suggesting unusual eating pattern), and low (<10, being within normal limits) score; for severity scale, a score ≥5 was considered as clinically significant. For the ineffectiveness item of the EDI-2, since the absence of standardized scores, three groups were arbitrarily established for statistical analysis: low (0–5), medium (6–10), and high (≥11) score.

### 2.4. Psychological Profile

All patients were evaluated by the same trained psychologist with a large experience of years of practice and a specific formation in eating disorders, through three one-hour semistructured interviews. During these interviews, depression, anxiety, phobias, and assertiveness were assessed by the following questionnaires: the Beck Depression Inventory II (BDI-II) [39]; the Spielberger State-Trait Anxiety Inventory (STAI) [40], form Y (French-Canadian adaptation IASTA-Y) [41]; the Rathus Assertiveness Schedule (RAS) [42], and the Marks and Mathews Fear Questionnaire (Fear M and M) [43]. These questionnaires were used to assess characteristic attitudes and symptoms of depression, anxiety, phobia, and assertiveness and not to perform a clinical diagnosis.

The following cutoff values were used for grouping total BDI-II scores: 0–9 not depressed; 10–15 mildly depressed; 16–24 moderately depressed; ≥25 severely depressed [44]. For the STAI-Y, considering the sex and the mean age of our cohort, a value ≥40 was applied to define clinically significant symptoms of transient and enduring levels of anxiety [45–47]. For the RAS, a score ≥105 was considered representative of a low degree of assertiveness, as proposed by Bouvard and Cottraux in the French version of the questionnaire [48]. The Fear M and M questionnaire has no formal cutoff point, so the following cutoff values were defined according to Cottraux [43] in the validated French version of the questionnaire: agoraphobia ≥27 and for social phobia ≥23.

### 2.5. Statistical Analysis

All analysis was performed using JMP 8 statistical package (SAS Institute, Cary, NC, USA). Results were expressed as number of patients and percentages or as mean ± standard deviation (SD). Between-group comparisons were performed using Chi-square for qualitative variables and Student's* t*-test or analysis of variance (ANOVA) for quantitative variables. The associations between anthropometric measurements, eating behaviours, and psychological profile were evaluated by univariate nonparametric Spearman's correlation. The associations between eating behaviours and psychological profile were further refined by Cochran-Armitage trend test and by multivariate forward stepwise regression (*P* value for entry is 0.05) using the scores of the psychological questionnaires (BDI-II, STAI-Y, and RAS), as dependent variables, and age, BMI, BITE score, and items score of EDI-2 as independent variables. The *R* ^2^ values for each final (i.e. including all significant variables) model were computed. Statistical significance was considered for *P* < 0.05.

## 3. Results

### 3.1. Patients' Characteristics

Of the 150 women, 12 (8%) were excluded from the analysis because of missing data for height (*n* = 3), waist circumference (*n* = 12), and/or STAI-Y (*n* = 9). The remaining 138 women had a mean age (±SD) of 41.4 ± 11.6 years, a mean BMI of 39.3 ± 6.4 kg/m^2^, and a mean waist circumference of 108.6 ± 14.3 cm.

One hundred and ten (80%) of all patients had made >5 intentional weight loss attempts and 109 (79%) presented with WCS. The BMI of patient with WCS was significantly higher than in patients without WCS: 40.3 ± 7 versus 35.8 ± 4 (*P* < 0.01).

### 3.2. Eating Profile

The mean BITE symptom and severity scores were 18.3 ± 6.4 and 4.0 ± 3.3, respectively. Almost half of the patients (48.6%) had a high (≥20) BITE symptom score, and 41% of them had a clinically significant BITE severity score. No correlations were found between the questionnaire scores and age or BMI or waist circumference of the patients. The mean scores of EDI-2's items were drive for thinness 8.9 ± 5.3; bulimia 5.2 ± 4.3; body dissatisfaction 20.8 ± 6.6; ineffectiveness 8.1 ± 6.9; perfectionism 5.7 ± 4.3; interpersonal distrust 4.0 ± 3.8; interoceptive awareness 7.6 ± 5.8; maturity fears 3.8 ± 4.1; asceticism 5.9 ± 3.2; impulse regulation 4.0 ± 4.4; social insecurity 5.6 ± 4.4.

### 3.3. Psychological Profile

The psychological profile of the patients is summarized in Table 1. Over half of the patients showed moderate (26%) or major (35%) depressive symptoms. Clinically significant signs of enduring levels of anxiety were found in 77% of patients, and a low degree of assertiveness was found in approximately half of the patients. Agoraphobia was identified in about 4% of patients and social phobia was identified in 20%. Conversely, no differences in BMI and waist circumference were found within all subclasses of the different psychological groups evaluated by the four questionnaires.

### 3.4. Association between Eating Behaviours and Psychological Profile

No correlations were found between psychological markers and age or BMI, while strong positive correlations were found between psychological markers and BITE components. Similarly, strong correlations were found between psychological markers and most EDI-2 components, namely, ineffectiveness, social insecurity, interoceptive awareness, and impulse regulation (Table 2).

The results of the stepwise regression analyses using psychological (BDI-II, STAI-Y State-Trait, and RAS) scores as dependent variables and the scores of BITE and EDI-2 items and age as independent variables are summarized in Table 3. Overall, depression and RAS were associated with BITE symptom score and EDI-2 ineffectiveness score, while anxiety was associated with BITE severity score and EDI-2 ineffectiveness score. In all models, EDI-2 ineffectiveness score was the variable most related with psychological scores, and in all models the percentage of variance explained was over 30%, with a value >50% for the Beck Depression score (Table 3).

**Figure 1:**
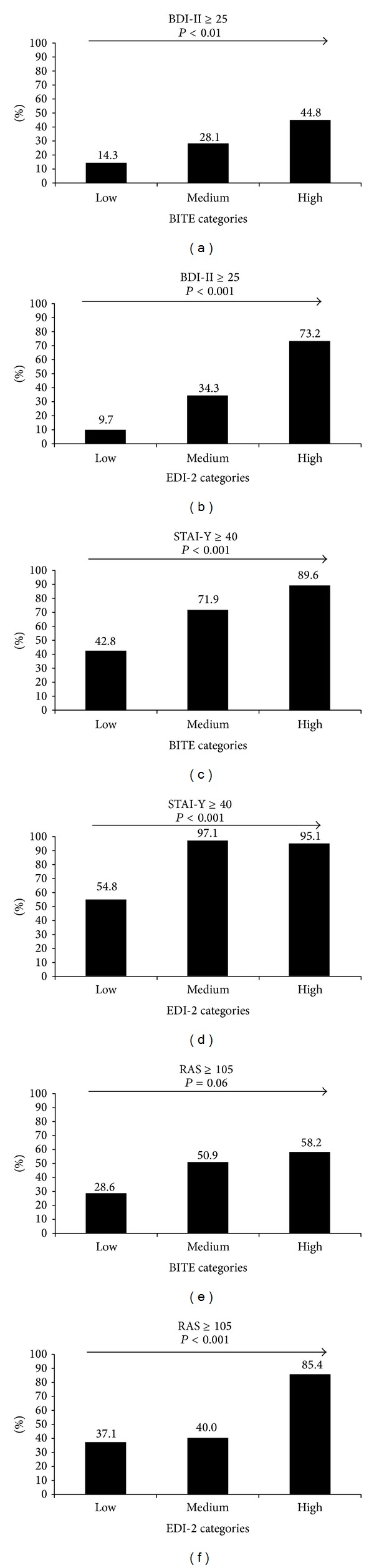
Psychological profile according to eating behaviour categories, as assessed by the Bulimic Investigatory Test of Edinburgh (BITE) symptoms score, and to ineffectiveness groups, as assessed by the Eating Disorder Inventory-2 (EDI-2). Results are expressed as percentage. BDI-II: Beck Depression Inventory II; STAI-Y: Spielberger State-Trait Anxiety Inventory, form Y; RAS: Rathus Assertiveness Schedule. Statistical analyses were performed using Cochran-Armitage trend test for qualitative variables and analysis of variance for quantitative variables.

**Figure 2:**
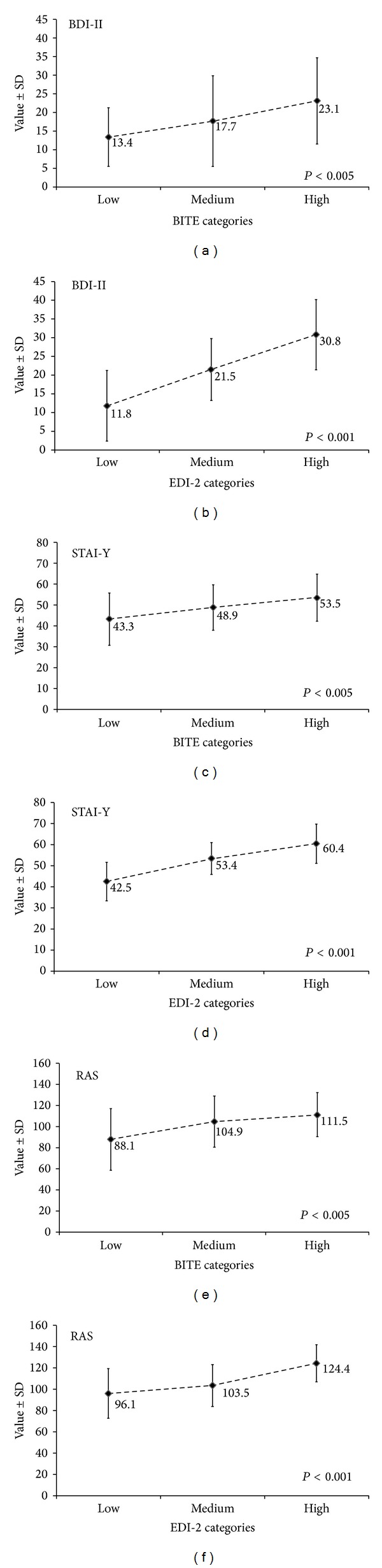
Psychological profile according to eating behaviour categories, as assessed by the Bulimic Investigatory Test of Edinburgh (BITE) symptoms score, and to ineffectiveness groups, as assessed by the Eating Disorder Inventory-2 (EDI-2). Results are expressed as mean ± standard deviation. BDI-II: Beck Depression Inventory II; STAI-Y, Spielberger State-Trait Anxiety Inventory, form Y; RAS: Rathus Assertiveness Schedule. Statistical analyses were performed using Cochran-Armitage trend test for qualitative variables and analysis of variance for quantitative variables.

The results of the different psychological questionnaires (BDI-II, STAI-Y State-Trait, RAS, and Fear M and M anxiety-depression and social phobia items) according to the BITE symptoms categories and EDI-2 ineffectiveness groups are summarized in Figures 1 and 2. Depression scores as well as the number of patients presenting with major depressive symptoms increased with high severity at BITE symptoms; similarly, STAI-Y Trait score and prevalence of patients presenting clinically significant symptoms of anxiety also increased with high severity of assertiveness. Finally, RAS scores increased with high severity of BITE symptoms while a borderline significant (*P* value <8%) trend was found for the prevalence of a high degree of the assertiveness (Figures 1 and 2).

## 4. Discussion

This study confirms that obese women with an obesity level 2 and seeking weight loss present high prevalence of typical psychological characteristics especially depressive and anxious symptoms and a low degree of assertiveness. These results are compatible with those shown in the literature [25, 49–56].

The prevalence and the severity of eating disorders and of psychological dysfunctions were not correlated with the degree and type of obesity. In a previous study, Onyike et al. [57] reported a positive correlation between major depression and obesity. However, they analyzed data from the Third National Health and Nutrition Examination Survey, which included underweight and obese subjects. Ahlberg et al. [58] also documented a significant correlation between depressive and anxious symptoms and abdominal distribution of body fat, but not with degree of obesity, and Hill and Williams [59] showed that a higher severity of the obesity was not bound with a higher frequency of psychological disorders.

On the other hand the prevalence and severity of depression, anxiety, and assertiveness increased according to the degree of eating disorders. Several studies showed that obese patients with BED presented with a higher prevalence and/or severity of most of psychological dysfunctions in comparison to obese patients without BED [26–32]. Didie and Fitzgibbon [60] showed that eating disorders account for psychological dysfunction independently of weight status and Fassino et al. [27, 28] showed that obese patients with eating disorders were at higher risk of being diagnosed with personality disorders and concluded that the presence of binge eating in obese women is a marker for greater medical and psychiatric morbidity. Particularly Behar et al. [61] showed that a lack of assertiveness was a significant trait in patients with eating disorders and may be considered as a predictive factor in the development of an eating disorder and Elfhag [62] showed that a lack of assertiveness characterized obese patients with more problematic eating behaviours and that a greater self-assertiveness was found in patients with a relatively more efficient eating strategy.

Furthermore Iliceto et al. [63] showed that overweight or obese women have higher scores on the EDI-2 subscales and Villarejo et al. [64] showed that obese women with BED had still higher scores on some EDI-2 subscales compare with obese women without BED or control group with normal weight. In another study Barry et al. [65] found also that patients with BED differ also in subscale of EDI-2. This variation was bound to the presence of BED but not with the obesity.

Stepwise regression analyses of eating status and psychological profile identified the ineffectiveness score of the EDI-2 as the major determinant of each of the questionnaires used (BDI-II, STAI-Y, and RAS). This is the most relevant and new finding of this work. In fact, for the first time, a potential predictive parameter for the identification of psychological distress has been recognized.

This finding has clinical implications in the health care of obese patients. Indeed, in order to recognize patients at risk of psychological dysfunctions, the first step in patient's evaluation should include a specific interview focusing on emotional behavioral aspects, such as body dissatisfaction, ineffectiveness, or perfectionism, for example. The development of such a questionnaire based on these emotional behavioral aspects could complement the interview and generate a promising data basis to conduct longitudinal cohort studies comparing results of long-term weight loss depending on different screening methods.

From a psychopathological point of view, these results (high ineffectiveness score, low interoceptive awareness, and impulsivity among obese) are in line with Hilde Bruch and Bergeret's psychoanalytical theories, as well as with Fairburn and Apfeldorfer's cognitive-behavioral models of the obese patient's psychological functioning. Fairburn's concept regarding the factors contributing to the persistence of eating disorders is of particular interest. He suggested that a biopsychological vulnerability, together with ambivalent relationships with the environment, results in low self-esteem. The eating disorder resulting from this low self-esteem attempts to compensate it, followed by a self-controlling attitude with restrictive eating behaviors, which provides an immediate feeling of control, thus positively reinforcing self-esteem. However this behavior cannot be maintained in the long term and inevitably results in loss of control (binging behaviors). The fragile self-esteem which had momentarily been established is then shattered and feelings of frustration, guilt, and inefficacy take over. This experience of failure produces feelings of anxiety, which remind us of the similarities between eating disorders and the phobic functioning described by Apfeldorfer.

The results of this study also highlight that psychiatric diagnosis per se might not be useful for outcome prediction and that identification of underlying psychological dynamics might be more promising. Recent critics with regard to DSM criteria for research purposes are in line with these assumptions. The fact that the underlying construct of “feeling of ineffectiveness” has been identified as clinically relevant by both, psychoanalytic and cognitive-behavioral theories, also adds to its potential value.

This study has some limitations. First of all it is a cross-sectional data. Secondly only women have been included and further research is needed to confirm the results in men. We have chosen to analyze depression, anxiety, phobias, and assertiveness by means of specific questionnaires, to facilitate an objective and standardized statistical evaluation, but without relying on DSM-IV diagnostic criteria for psychiatric disorders; the lack of psychiatric diagnoses therefore hampers an evaluation of their predictive value. The arbitrarily established groups for the ineffectiveness item of the EDI-2 lead also to a limitation and a study for standardized scores should be done. Finally, this outpatient obesity clinic is a tertiary centre for eating disorders and obesity and we may therefore assume that the studied population consists of the most severe cases. In future study with a greater number of patients or patients of different types should be done to confirm these results

## 5. Conclusions

In conclusion this study confirms the high prevalence of psychological dysfunction such as depression, anxiety, self-affirmation, and phobia in obese women trying to lose weight and that their severity is correlated to the degree of eating disorders. In addition, the fact that the feeling of ineffectiveness constitutes the major determinant of these patients' psychological profiles was demonstrated. This evidence opens new ways for the development of screening tools for outcome prediction in the field of eating and psychological disorders and for early and targeted intervention for patients at risk for unfavorable developments after bariatric surgery. For example, the future development of easy questionnaires based on these parameters done by patients alone at home will be a great development and a great benefit in time and in money for a better care of these patients.

## Figures and Tables

**Table 1:** Distribution and characteristics of patients according to psychological profile.

	Score	N° patients (%)	Age (years)	BMI (kg/m^2^)
BDI-II	0–9	37 (26.8)	40.9 ± 11.4	39.5 ± 6.7
	10–15	17 (12.3)	45.2 ± 11.6	39.0 ± 8.6
	16–24	36 (26.1)	40.7 ± 10.2	39.8 ± 6.5
	≥25	48 (34.8)	40.9 ± 12.7	38.9 ± 5.3
Test (*P* value)			0.98 (0.40)	0.10 (0.96)
STAI-Y State	≤39	48 (34.8)	40.8 ± 9.8	39.2 ± 7.3
	≥40	90 (65.2)	41.7 ± 12.4	39.3 ± 5.9
Test (*P* value)			0.19 (0.67)	0.01 (0.91)
STAI-Y Trait	≤39	31 (22.5)	42.0 ± 10.8	39.2 ± 6.6
	≥40	107 (77.5)	41.2 ± 11.8	39.3 ± 6.3
Test (*P* value)			0.12 (0.73)	0.01 (0.96)
RAS	<105	66 (47.8)	41.8 ± 11.4	40.2 ± 7.2
	≥105	72 (52.2)	41.1 ± 11.8	38.4 ± 5.3
Test (*P* value)			0.31 (0.76)	1.49 (0.14)
Fear M and M				
Agoraphobia	≤26	133 (96.4)	41.6 ± 11.5	39.3 ± 6.4
	≥27	5 (3.6)	36.8 ± 13.7	40.3 ± 4.2
Test (*P* value)			0.82 (0.37)	0.13 (0.71)
Social phobia	≤22	111 (80.4)	42.5 ± 11.5	39.5 ± 5.0
	≥23	27 (19.6)	37.0 ± 11.0	38.3 ± 5.0
Test (*P* value)			4.93 (0.03)	0.79 (0.38)

Results are expressed as number of subjects and percentages or as mean ± standard deviation. BMI: body mass index; BDI-II: Beck Depression Inventory II; STAI-Y: Spielberger State-Trait Anxiety Inventory, form Y; RAS: Rathus Assertiveness Schedule; Fear M and M: Marks and Mathews Fear Questionnaire. Statistical analyses performed using Student's *t*-test or analysis of variance for quantitative variables.

**Table 2:** Spearman's correlations between anthropometric parameters, eating behaviour, and psychological profile.

	BDI-II	STAI-Y State	STAI-Y Trait	RAS
Age	−0.102^NS^	−0.084^NS^	−0.150^NS^	0.026^NS^
BMI	−0.003^NS^	0.041^NS^	−0.005^NS^	−0.104^NS^
Waist circumference	0.027^NS^	−0.166^NS^	−0.008^NS^	0.008^NS^
BITE				
Symptom	0.374***	0.398***	0.338***	0.275***
Severity	0.261**	0.314***	0.283***	0.184*
EDI-2				
Ineffectiveness	0.677***	0.616***	0.709***	0.548***
Interpersonal distrust	0.221**	0.088^ NS^	0.333***	0.234**
Desire for thinness	0.291***	0.375***	0.294***	0.133^ NS^
Bulimia	0.335***	0.292***	0.285***	0.322***
Body dissatisfaction	0.105^ NS^	0.250**	0.251**	0.200*
Perfectionism	0.190*	0.218*	0.162^ NS^	0.082^ NS^
Interoceptive awareness	0.564***	0.486***	0.488***	0.438***
Maturity fears	0.304***	0.265**	0.360***	0.115^ NS^
Asceticism	0.260**	0.366***	0.262**	0.232**
Impulse regulation	0.461***	0.387***	0.472***	0.315***
Social insecurity	0.576***	0.462***	0.565***	0.437***

BMI: body mass index; BITE: Bulimic Investigatory Test of Edinburgh; EDI-2: Eating Disorder Inventory-2; BDI-II: Beck Depression Inventory II; STAI-Y: Spielberger State-Trait Anxiety Inventory, form Y; RAS: Rathus Assertiveness Schedule. Statistical analysis by Spearman's rank correlations: ^NS^not significant; *P < 0.05; **P < 0.01; ***P < 0.001.

**Table 3:** Multivariate analysis of the associations between eating behaviours and psychological profile.

	BDI-II	STAI-Y State	STAI-Y Trait	RAS
Age	—	—	—	0.150
BITE				
Symptom	0.222	—	—	0.190
Severity	—	0.188	0.199	—
EDI-2				
Ineffectiveness	0.653	0.636	0.651	0.495
Interpersonal distrust	—	−0.149	—	—
**R** ^2^ ** of model**	**0.546**	**0.400**	**0.478**	**0.323**

Statistical analysis by forward stepwise linear regression. BITE: Bulimic Investigatory Test of Edinburgh; EDI-2: Eating Disorder Inventory-2; BDI-II: Beck Depression Inventory II; STAI-Y: Spielberger State-Trait Anxiety Inventory, form Y; RAS: Rathus Assertiveness Schedule.

Results are expressed as standardized slope. —: not retained. The other items of the EDI-2 (desire for thinness, bulimia, body dissatisfaction; perfectionism, interoceptive awareness, maturity fears, asceticism, impulse regulation, and social insecurity) were not retained.
